# Steroid Receptor Coactivator‐1 Drives Tumor‐Associated Macrophage Reprogramming by Mediating MMP12 Transcription in Pancreatic Cancer Perineural Invasion

**DOI:** 10.1002/advs.202416575

**Published:** 2025-09-23

**Authors:** Ke Cheng, Liang Liu, Miaomiao Gong, Yuke Ji, Chunmei Bai, Xiangqian Guo, Hui Chen, Jinjin Pan, Ying Zhang, Yuhui Yuan

**Affiliations:** ^1^ The Second Affiliated Hospital Institute of Cancer Stem Cell Dalian Medical University Dalian 116044 China; ^2^ Department of Pharmacology and Chemical Biology Key Laboratory of Cell Differentiation and Apoptosis of Chinese Ministry of Education Shanghai Jiao Tong University School of Medicine. Collaborative Innovation Center for Clinical and Translational Science by Chinese Ministry of Education & Shanghai 280 South Chongqing Road Huangpu Shanghai 200025 China; ^3^ Institute of Biomedical Informatics Bioinformatics Center Henan Provincial Engineering Center for Tumor Molecular Medicine School of Software School of Basic Medical Sciences Academy for Advanced Interdisciplinary Studies Henan University Kaifeng 47500 China; ^4^ Sixth Department of Liver Disease Dalian Public Health Clinical Center Dalian 116044 China

**Keywords:** pancreatic cancer, perineural invasion, steroid coactivator receptor‐1, tumor associated macrophages

## Abstract

Perineural invasion (PNI) is a hallmark of pancreatic cancer aggressiveness. However, the feasibility of manipulating tumor‐associated macrophage (TAM) reprogramming to influence PNI development remains unclear. Methods: Using in vitro (tumor‐DRG co‐culture) and in vivo (sciatic nerve injection) models coupled with protein identification by liquid chromatography‐mass spectrometry (LC‐MS) and ChIP‐seq, the role of steroid receptor coactivator‐1 (SRC‐1) is investigated in TAM reprogramming and PNI. SRC‐1 is up‐regulated in TAMs and promotes PNI by binding to signal transducer and activator of transcription 1(STAT1) to enhance matrix metallopeptidase 12 (MMP12) transcription. SRC‐1 knockdown attenuated M2‐like characteristics in TAMs, reduced MMP12 secretion, and suppressed PNI. Importantly, blocking the SRC‐1/STAT1/MMP12 axis (using SRC‐1‐KO TAMs or MMP12 inhibitors) attenuated PNI progression in vivo. SRC‐1 reprograms TAMs via STAT1‐mediated MMP12 activation to facilitate PNI. Targeting SRC‐1 disrupts this axis and presents a novel therapeutic strategy against PNI in pancreatic cancer.

## Graphical abstract

Steroid receptor coactivator‐1 (SRC‐1) reprograms tumor‐associated macrophages (TAMs) via signal transducer and activator of transcription 1(STAT1)‐mediated matrix metallopeptidase 12 (MMP12) transcription to drive perineural invasion (PNI) in pancreatic cancer. Mechanistically, SRC‐1 binds STAT1 to enhance MMP12 promoter activity, boosting MMP12 secretion. This axis promotes TAM M2 polarization, tumor cell invasion, and neural infiltration. Targeting SRC‐1 or MMP12 attenuates PNI progression, revealing a novel therapeutic strategy against this aggressive hallmark.

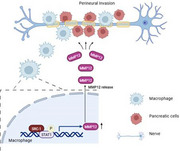

## 1 Introduction

Pancreatic cancer, the third leading cause of cancer‐related deaths,^[^ ^1^ ^]^ is characterized by perineural invasion (PNI) in >80% of patients. PNI causes debilitating neuropathic pain and serves as an independent predictor of poor survival and recurrence^[^ ^2^ ^]^; however, its driving factors have not been comprehensively elucidated. Tumor‐associated macrophages (TAMs) are enriched at PNI sites, where they secrete glial cell line‐derived neurotrophic factor (GDNF) to promote cancer‐nerve crosstalk ^[^ ^3^ ^]^, making TAM reprogramming a clinically promising anti‐PNI strategy.^[^ ^4^, ^5^ ^]^

Although macrophages shift from anti‐tumor M1 to pro‐tumor M2 phenotypes during cancer progression,^[^ ^6^ ^]^ the master regulators controlling this switch in the context of PNI are undefined. Owing to its role in macrophage recruitment and autophagy, steroid receptor coactivator‐1 (SRC‐1) has emerged as a critical candidate.^[^ ^7^, ^8^ ^]^ In addition, we previously found that SRC‐1 deletion suppresses metastasis without affecting primary tumors.^[^ ^9^ ^]^ Importantly, we hypothesized that SRC‐1 reprogrammed TAMs via signal transducer and activator of transcription (STAT1) to activate matrix metallopeptidase 12 (MMP12), a macrophage‐derived metalloprotease implicated in nerve remodeling ^[^ ^10^ ^]^, thereby bridging the transcriptional regulation of PNI pathogenesis.

Here, we defined the SRC‐1/STAT1/MMP12 axis as a mechanistic link. SRC‐1 binds to STAT1 to enhance MMP12 promoter activity, driving TAM‐mediated PNI. This study elucidates how targeting this axis attenuates PNI in vivo, offering novel therapeutic avenues against PNI in pancreatic cancer.

## 2 Result

### 2.1 Manipulating the Expression Level of SRC‐1 Can Impact TAM Reprogramming

**Figure 1:**
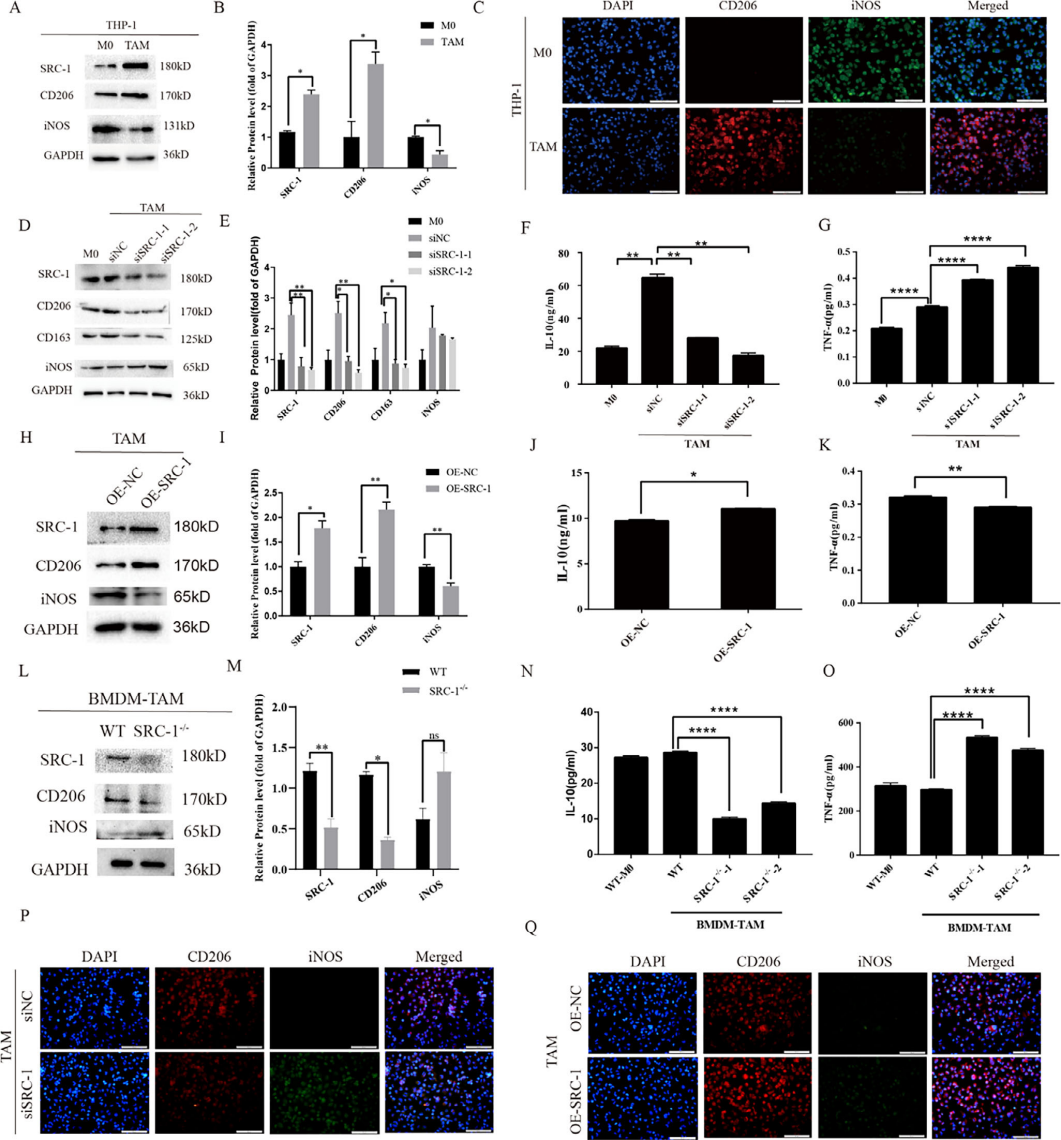
SRC‐1 regulates the reprogramming of TAMs. A, B) Western blot analysis of SRC‐1, CD206, and iNOS expression in M0 and TAMs. n = 3. Significance was calculated with Student's *t*‐test.^*^ *P<*0.05,^**^ *P<*0.01,^***^ *P<*0.001.C) Immunofluorescence staining showing CD206 and iNOS expression in M0 derived from THP‐1 cells and TAMs. D, E) Western blot analysis of SRC‐1, CD206, CD163, and iNOS expression in indicated groups of TAMs.n = 3. Significance was calculated with one‐way ANOVA with Tukey post‐test. ^*^ *P<*0.05, ^**^ *P<*0.01, ^***^ *P<*0.001. F, G, J, K, N, O) ELISA quantification of IL‐10 and TNF‐α levels in CM from TAMs and BMDMs across specified groups.n = 3. Student's *t*‐test was used to compare the two groups, and one‐way ANOVA with Tukey post‐test was used for the rest. ^*^ *P<*0.05, ^**^ *P<*0.01, ^***^ *P<*0.001. H, I) Western blot analysis of SRC‐1, CD206, and iNOS expression in TAMs after SRC‐1 over‐expression. n = 3. Significance was calculated with Student's *t*‐test. ^*^ *P<*0.05, ^**^ *P<*0.01, ^***^ *P<*0.001. L, M) Western blot analysis of SRC‐1, CD206, and iNOS expression in BMDMs from WT and SRC‐1^‐/‐^ mice. n = 3. Significance was calculated with Student's *t*‐test. ^*^ *P<*0.05, ^**^ *P<*0.01, ^***^ *P<*0.001, ns, no significant difference. P, Q) Immunofluorescence staining of CD206 and iNOS expression in different groups of TAMs.

Human monocytic THP‐1 cells were induced to differentiate into macrophages (M0) using phorbol 12‐myristate‐13‐acetate (PMA), followed by polarization into TAMs using conditioned medium (CM) from pancreatic cancer cells. Western blot analysis revealed higher levels of SRC‐1 and the M2 marker CD206 in TAMs than in M0 macrophages; the expression of the M1 macrophage marker iNOS appeared to decrease,(**Figure** **1**A,B). Immunofluorescence staining further confirmed elevated CD206 expression in TAMs relative to M0 cells, with relatively low iNOS expression (Figure 1C). These experiments indicated significantly higher expression levels of SRC‐1 and M2 markers in TAMs. To further explore the role of SRC‐1 role in TAM reprogramming, we used small interfering RNA (siRNA) to knockdown SRC‐1 expression in THP‐1 cells. The results showed that SRC‐1 knockdown reduced the expression of M2 markers CD206 and CD163 in TAMs (Figure 1D,E). ELISA results demonstrated decreased secretion of the M2‐associated cytokine IL‐10 with SRC‐1 knockdown, coupled with increased secretion of the M1‐associated cytokine TNF‐α (Figure 1F,G). Conversely, over‐expression of SRC‐1 led to up‐regulation of CD206 (Figure 1H,I), increased IL‐10 secretion, and decreased TNF‐α secretion (Figure 1J,K). To validate these findings, primary bone marrow‐derived macrophages (BMDM) from wild‐type (WT) and SRC‐1^‐/‐^ mice were stimulated with CM from KRAS/p53^m/+^ pancreatic cancer cells, showing trends similar to those of human‐derived macrophages. Similar results were observed for BMDMs (Figure 1L–O). Immunofluorescence staining further confirmed that siSRC‐1 reduced CD206 expression compared to the siNC group, while increasing iNOS expression. Conversely, OE‐SRC‐1 increased CD206 expression relative to the OE‐NC group (Figure 1P,Q). These findings suggest that the knockdown of SRC‐1 in TAMs suppresses their M2‐polarized phenotype.

### 2.2 SRC‐1 Modulates PNI in Pancreatic Cancer by Reprogramming TAM

**Figure 2:**
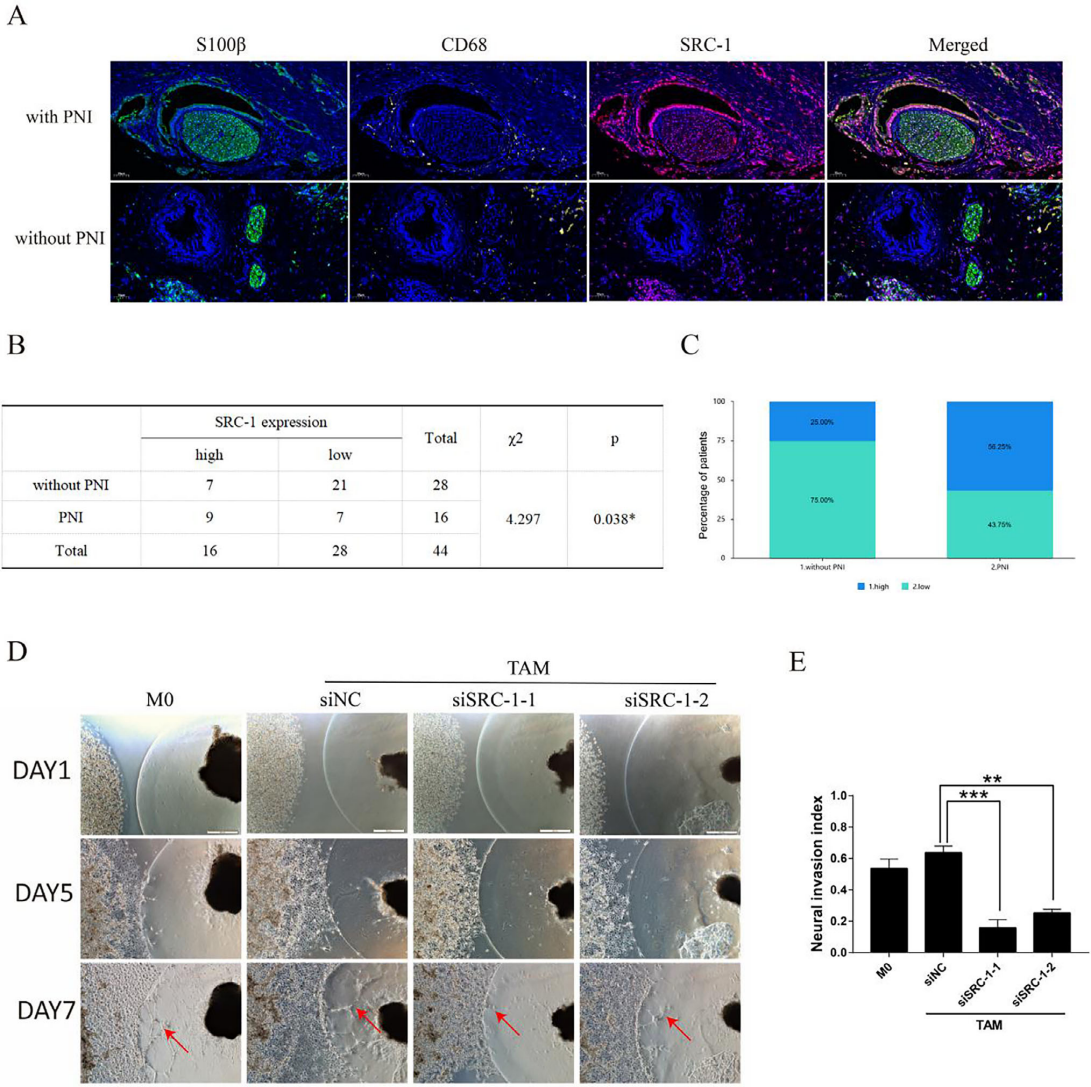
SRC‐1 modulates PNI in pancreatic cancer by reprogramming TAM. A) Representative images of IF staining for SRC‐1 (red), S100 (green), and CD68 (yellow) in tissue microarray. S100 was used as a neuronal cell marker, and CD68 was used as a macrophage marker. Scale bars, 50 µm. B) The relationship between PNI and SRC‐1 expression was analyzed. n = 44. *P* values were calculated using Fisher's exact test. ^*^ *P* < 0.05. C) SRC‐1 expression levels of the “without PNI” group and “with PNI group” were compared using the immunoreactive score. D) The changes in nerve invasion index were observed after the addition of CM from different TAM groups to the in vitro PNI model. The red arrow indicates the leading edge of tumor cell invasion. Scale bar: 500 µm. E) Quantitative analysis of the neural invasion index.n = 3. Significance was calculated with one‐way ANOVA with Tukey post‐test.^**^ *P* < 0.01, ^***^ *P* < 0.001.

To investigate whether SRC‐1 is a key factor in regulating macrophages in PNI in pancreatic cancer, we employed a tissue microarray to analyze the association between SRC‐1 and CD68 expression and the PNI status in tumor tissue. Tissue microarrays from pancreatic cancer patients (n = 44) were classified into two groups by experienced pathologists: 36% of the patients (n = 16) were diagnosed with the severe PNI group, and the others (n = 28) were categorized as the “without PNI group.” We performed IF multi‐staining for CD68 and SRC‐1 in tissue microarrays to evaluate the relationship between SRC‐1‐reprogrammed‐TAMs and PNI in pancreatic cancer. Abundant macrophages and high SRC‐1 expression in CD68^+^ cells were observed in patients with pancreatic cancer in the “with PNI group,” whereas the expression of SRC‐1 in CD68^+^ cells was comparatively low in the “without PNI group” (**Figure** **2**A). This result indicates that the expression of SRC‐1 is strongly associated with the PNI status of the tumors. In the “without PNI group,” the high expression of SRC‐1 in CD68^+^ cells was 25.00% (7/28), and 56.25% (9/16) in the “with PNI group” (Figure 2B). Our analysis revealed that the expression of SRC‐1 in the macrophages was strongly associated with PNI (Figure 2C). We hypothesized that SRC‐1 reprogramming of macrophages could further influence PNI in pancreatic cancer. Initially, we established an in vitro PNI model by co‐culturing mouse dorsal root ganglia (DRG) with tumor cells. We evaluated the severity of PNI using a nerve invasion index (where the total length between tumor cells and DRG is defined as β, and the distance migrated by tumor cells toward DRG as α; α/β represents the nerve invasion index). The addition of CM from TAM‐siNC and TAM‐siSRC‐1 to the in vitro PNI model revealed that tumor cells in the siNC group made synaptic contact with the DRG by the seventh day of the co‐culture. Conversely, in the siSRC‐1 group, the distance of tumor cell migration toward the nerves was significantly reduced, showing a marked difference in the nerve invasion index (Figure 2D,E). Collectively, these results suggest that SRC‐1 influences the progression of PNI in pancreatic cancer through reprogramming macrophages.

### 2.3 SRC‐1 Modulates Tumor Cell Behavior via TAM Reprogramming in Pancreatic Cancer

**Figure 3:**
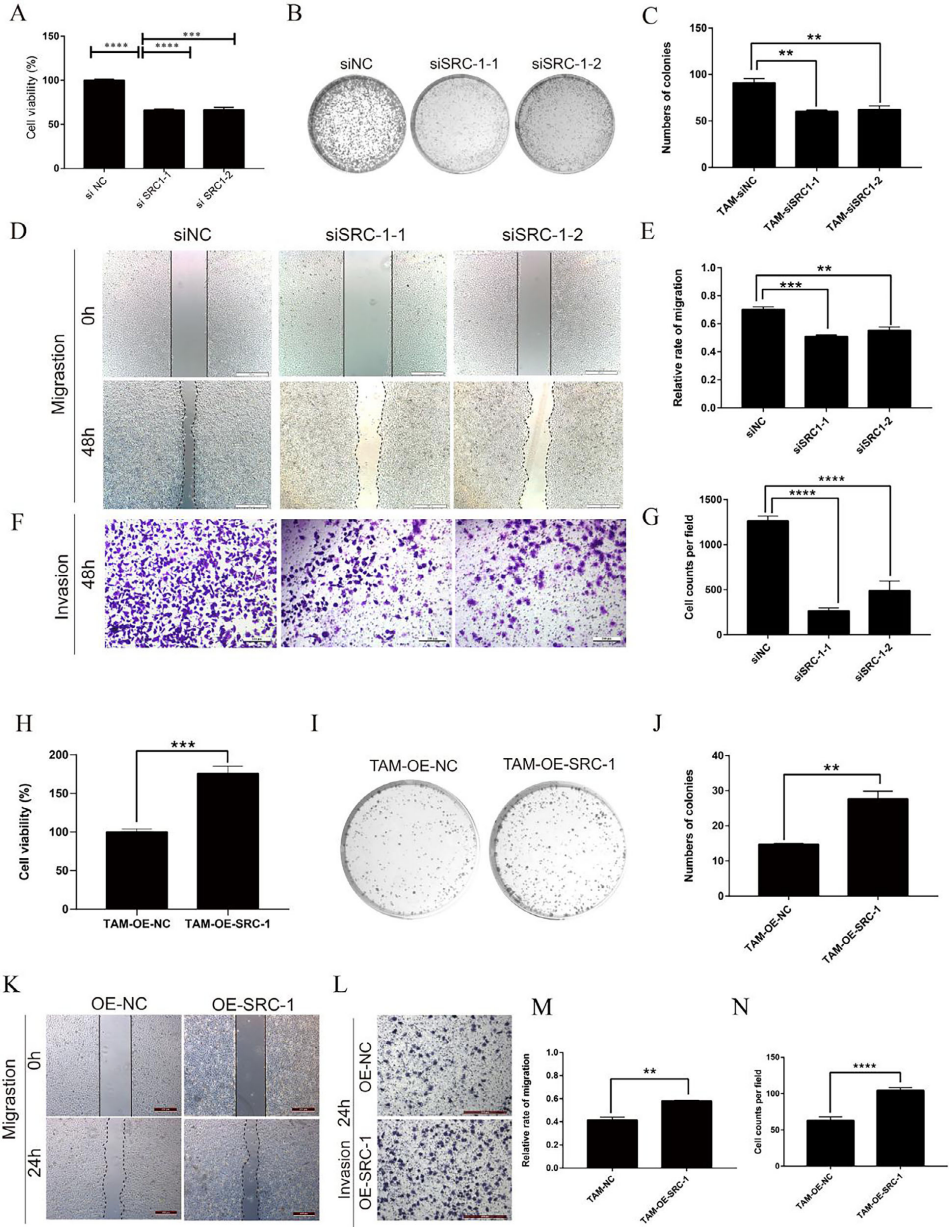
SRC‐1 regulates tumor cell behavior in pancreatic cancer PNI via macrophage reprogramming. A, H) CCK8 assays were performed to evaluate the proliferation capacity of tumor cells cultured with CM from different types of TAMs. n = 3. Student's *t*‐test was used to compare the two groups, and one‐way ANOVA with Tukey post‐test was used for the rest. ^***^ *P<*0.001, ^****^ *P<*0.0001. B, I) Colony formation assays were performed to assess the clonogenic ability of tumor cells cultured with CM from different types of TAMs. C, J) Quantification of the colony formation assays. n = 3. Student's *t*‐test was used to compare the two groups, and one‐way ANOVA with Tukey post‐test was used for the rest. ^*^ *P<*0.05, ^**^ *P<*0.01, ^***^ *P<*0.001. D, K) Wound healing assays to examine the migratory ability of tumor cells cultured with CM from different types of TAMs. Scale bar: 500 µm. E, M) Quantification of the wound healing assays. n = 3. Student's *t*‐test was used to compare the two groups, and one‐way ANOVA with Tukey post‐test was used for the rest. ^*^ *P<*0.05, ^**^ *P<*0.01, ^***^ *P<*0.001. F, L) Trans‐well assays to measure the invasive capability of tumor cells cultured with CM from different types of TAMs. Scale bar: 200 µm. G, N) Quantification of the Tran‐swell assays. n = 3. Student's *t*‐test was used to compare the two groups, and one‐way ANOVA with Tukey post‐test was used for the rest.^*^ *P<*0.05, ^**^ *P<*0.01, ^***^ *P<*0.001.

Next, we postulated that SRC‐1‐driven TAM reprogramming modulates key hallmarks of tumor malignancy (e.g., proliferation/invasion), which in turn mediates PNI progression. To test this hypothesis, we collected the CM from TAM‐siNC and TAM‐siSRC‐1 cells. The results of the CCK8 assay showed substantially reduced proliferation of tumor cells in the TAM‐siSRC‐1 group than that in the TAM‐siNC group (**Figure** **3**A). Colony formation assays demonstrated weaker clonogenic ability in the TAM‐siSRC‐1 group than in the TAM‐siNC group (Figure 3B,C). Wound healing assays revealed a decreased migratory capacity of tumor cells in the TAM‐siSRC‐1 group relative to the TAM‐siNC group (Figure 3D,E). Transwell assays indicated a poorer invasive capability of tumor cells in the TAM‐siSRC‐1 group than in the TAM‐siNC group (Figure 3F,G). Conversely, when SRC‐1 was overexpressed in TAMs, the opposite results were observed (Figure 3H,N). Collectively, these findings suggest that SRC‐1 may influence the proliferation, migration, and invasion abilities of tumor cells through macrophage modulation, thereby affecting PNI in pancreatic cancer.

### 2.4 SRC‐1 Deficiency in TAMs Attenuates Pancreatic Cancer PNI in Mouse Model

**Figure 4:**
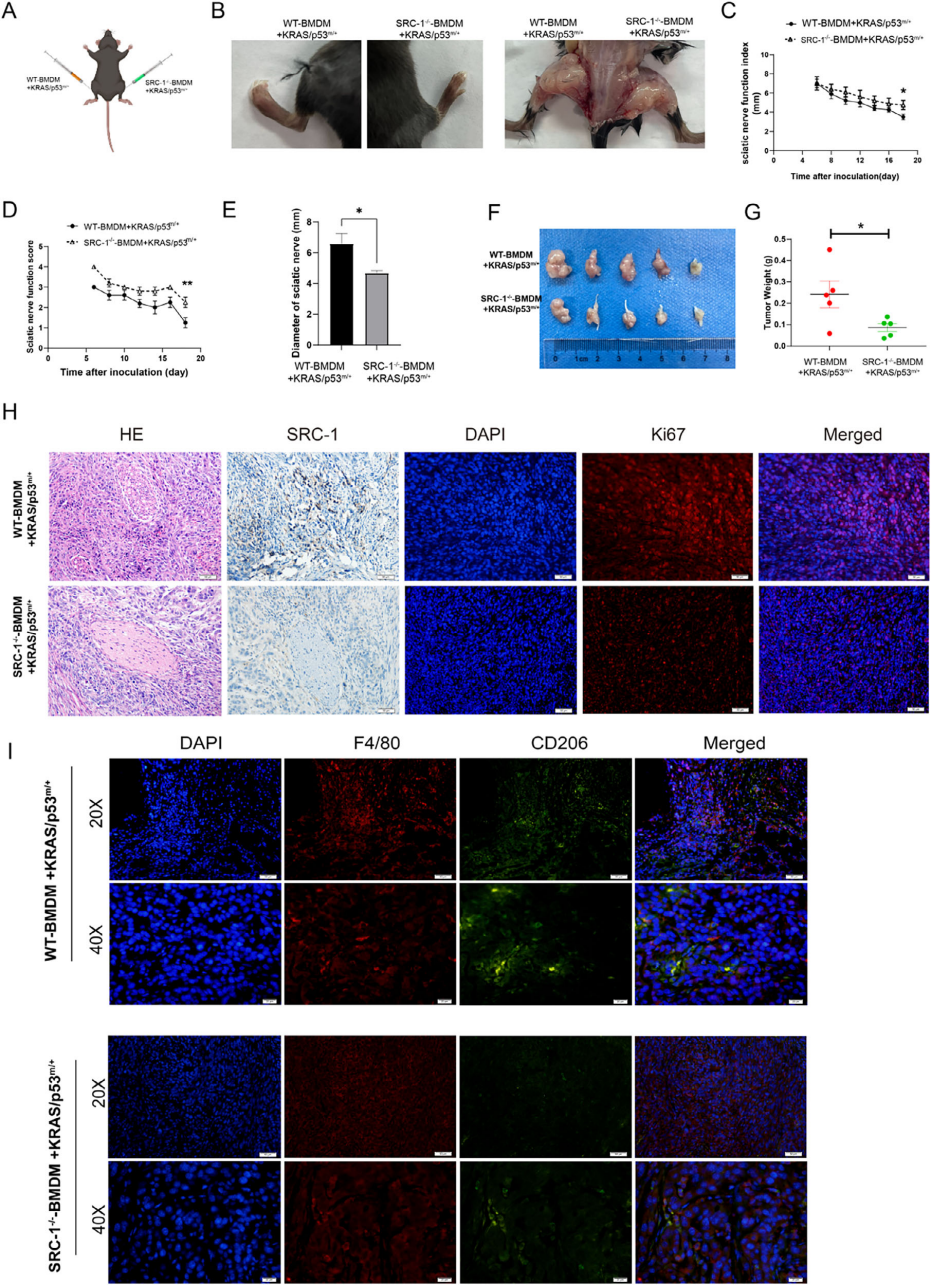
Reduced PNI in pancreatic cancer by SRC‐1‐deficient TAMs in a mouse model. A) WT‐BMDM and SRC‐1^‐/‐^BMDM mixed with KRAS/p53^/m/+^ cells were injected into the left and right sciatic nerves of mice, respectively. (n = 5) B) Mice were euthanized and dissected for imaging 21 days post‐injection. C, D) Sciatic nerve function index and scoring were recorded starting 6 days post‐injection, graded based on hindlimb response to manual extension into normal, mildly affected, severely affected, and completely paralyzed categories. n = 5. Student's *t*‐test was performed to compare the two groups at the final time point. ^*^ *P<*0.05, ^**^ *P<*0.01. E) Measurement of sciatic nerve diameter in WT‐BMDM and SRC‐1^‐/‐^BMDM groups. n = 5. Significance was calculated with Student's *t*‐test. ^*^ *P<*0.05. F, G) Tumor size and weight measurement in WT‐BMDM and SRC‐1^‐/‐^BMDM groups. n = 5 per group. Significance was calculated with Student's *t*‐test. ^*^ *P* < 0.05. (H, I) Hematoxylin and Eosin (H＆E) staining, immunohistochemistry and immunofluorescence staining detecting expression of SRC‐1, Ki67, F4/80, and CD206 in WT‐BMDM and SRC‐1^‐/‐^BMDM groups.

To further validate our in vitro findings, primary BMDMs were isolated from WT and SRC‐1^‐/‐^ mice and polarized into TAMs using CM from KRAS/p53^m/+^ spontaneous pancreatic cancer cells. Five 5–6‐week‐old C57 mice were used per group. WT‐BMDMs (1 × 10^5^) and KRAS/p53^m/+^ cells (3 × 10^4^) were injected into the left sciatic nerve, whereas SRC‐1^‐/‐^ BMDMs (1 × 10^5^) and KRAS/p53^m/+^ cells (3 × 10^4^) were injected into the right sciatic nerve (**Figure** **4**A). Nerve function indices and scores were recorded starting from day 6 post‐injection, with the mice euthanized on day 21 for dissection. Results indicated more pronounced nerve expansion owing to tumor cell infiltration in the WT‐BMDM group than the SRC‐1^‐/‐^ BMDM group (Figure 4B). Paralysis was observed in WT‐BMDM‐injected mice, whereas partial paralysis was noted in SRC‐1^‐/‐^ BMDM‐injected mice. Additionally, both nerve function indices and scores were lower in the WT‐BMDM group compared to the SRC‐1^‐/‐^ BMDM group (Figure 4C,D). Furthermore, nerve diameter and tumor weight were greater in the WT‐BMDM group than in the SRC‐1^‐/‐^ BMDM group (Figure 4E–G). These results collectively suggest that WT‐TAMs exhibit a stronger promotion of PNI in vivo than SRC‐1^‐/‐^ TAMs. Immunohistochemistry and immunofluorescence staining revealed higher expression levels of SRC‐1 and Ki67 in the tumor regions of WT‐BMDM‐injected mice than in those of SRC‐1^‐/‐^ BMDM‐injected mice (Figure 4H), indicating the enhanced proliferative capacity of tumor cells in the WT‐BMDM group. To confirm the role of SRC‐1 in TAM reprogramming in vivo, immunofluorescence analysis was performed to evaluate CD206 expression. The results showed reduced CD206 expression in the tumor areas of SRC‐1^‐/‐^ BMDM‐injected mice, supporting the notion that downregulation of SRC‐1 leads to a shift in the TAMs polarization state.

### 2.5 TAMs Reprogrammed by SRC‐1 Secretion MMP12 Induce PNI

**Figure 5:**
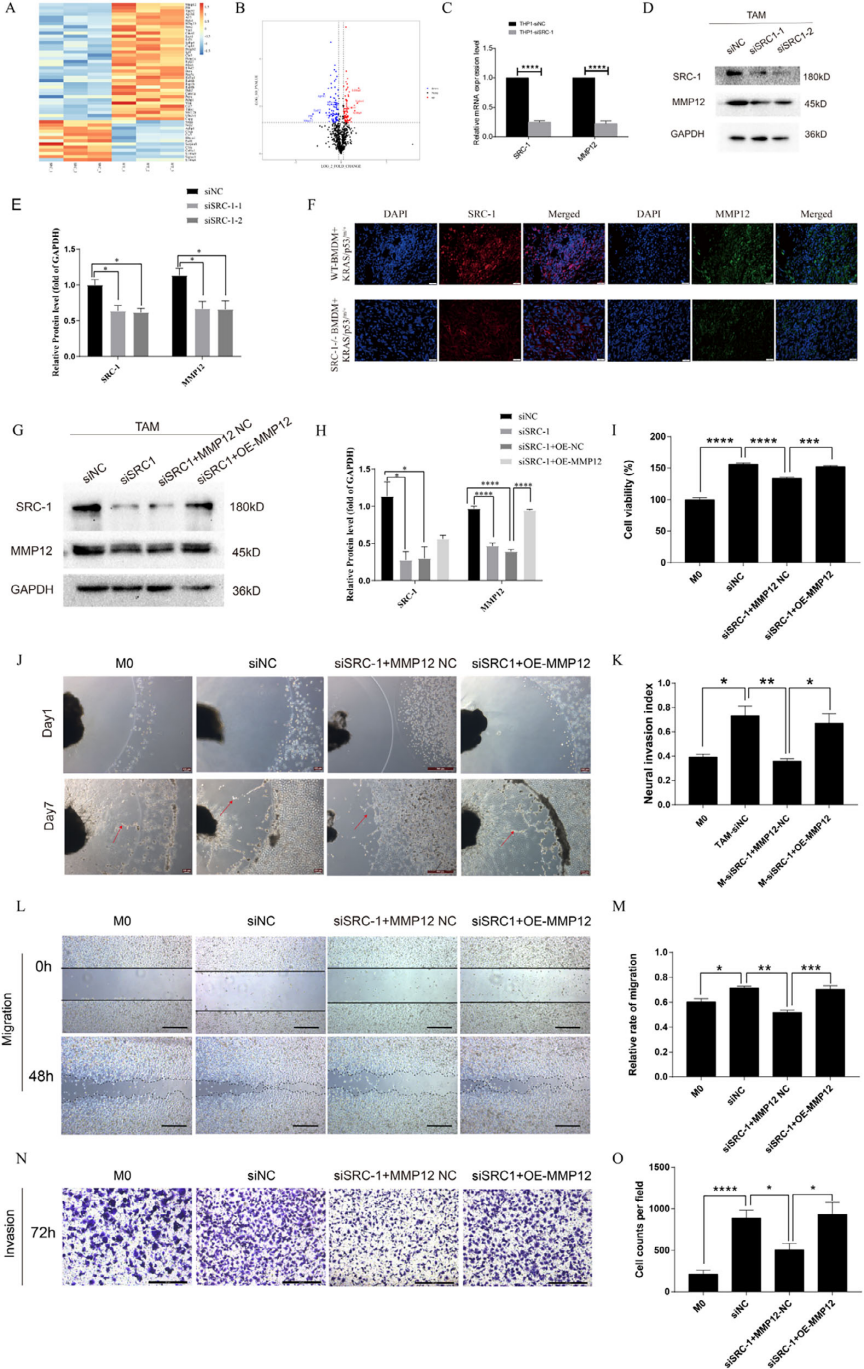
TAMs reprogrammed by SRC‐1 secretion MMP12 induce PNI. A) BMDMs were induced into TAMs from WT and SRC‐1^‐/‐^ mice, and CM was collected for differential protein analysis by mass spectrometry (n = 3). B) Volcano plot of differentially expressed proteins between WT and SRC‐1^‐/‐^ groups. C) qPCR analysis revealed decreased MMP12 mRNA levels upon SRC‐1 downregulation.n = 3. Significance was calculated with Student's *t*‐test. ^****^ *P<*0.0001. D, E) Western blotting detected the reduced MMP12 protein expression following SRC‐1 down‐regulation. n = 3. Significance was calculated with one‐way ANOVA with Tukey post‐test.^*^ *P<*0.05. F) Immunofluorescence staining to detect the expression of MMP12 in mouse tissues from the sciatic nerve injection models. G,H) Western blot confirmation demonstrated successful SRC‐1 knockdown and MMP12 over‐expression. n = 3. Significance was calculated with one‐way ANOVA with Tukey post‐test. ^*^ *P<*0.05, ^****^ *P<*0.0001. I) CCK8 assays were utilized to indicate the enhanced proliferation of AsPC‐1 cells upon SRC‐1 down‐regulation and MMP12 over‐expression in TAMs. n = 3. Significance was calculated with one‐way ANOVA with Tukey post‐test.^***^ *P<*0.001,^****^ *P<*0.0001. J) Rescue experiments assessing changes in in vitro PNI following SRC‐1 down‐regulation and MMP12 over‐expression. The red arrow indicates the leading edge of pancreatic cancer cell invasion. K) The neural invasion index of the rescue experiments. n = 3. Significance was calculated with one‐way ANOVA with Tukey post‐test. ^*^ *P<*0.05, ^**^ *P<*0.01. L–O) Wound healing and Trans‐well assays demonstrated the increased migration and invasion ability of AsPC‐1 cells upon SRC‐1 down‐regulation and MMP12 over‐expression in TAMs.n = 3. For wound healing assays: the scale bar is 500 µm. For the Transwell assay, the scale bar is 400 µm.n = 3. Significance was calculated with one‐way ANOVA with Tukey post‐test. ^*^ *P* <0.05, ^**^ *P* < 0.01, ^***^ *P* < 0.001, ^****^ *P* < 0.0001.

To investigate the specific factors underlying the altered tumor‐regulatory capability of TAMs under SRC‐1 knockdown conditions, we isolated BMDMs from WT and SRC‐1^‐/‐^ mouse bone marrow and induced them to form TAMs. We compared the protein expression profiles of WT‐BMDMs and SRC‐1^‐/‐^ BMDMs. The results indicate that MMP12 exhibited the most significant change in protein intensity relative to SRC‐1^‐/‐^ and WT (**Figure** **5**A,B). Subsequent validation in THP‐1 cells confirmed the stable reduction of MMP12 mRNA upon SRC‐1 knockdown (Figure 5C). This was further corroborated by western blotting, which showed a decrease in MMP12 protein levels following SRC‐1 knockdown (Figure 5D,E). Simultaneously, in the sciatic nerve injection mouse model that the expression of MMP12 in the SRC‐1^‐/‐^ group was lower than that in the WT group (Figure 5F).

Matrix metalloproteinases (MMPs) are typically associated with extracellular matrix (ECM) degradation and remodeling, suggesting their potential impact on tissue structure and function.^[^ ^11^ ^]^ Recently, the critical roles of MMPs in various biological processes, particularly in cancer metastasis, have been increasingly recognized.^[^ ^12^ ^]^ Among them, MMP12, also known as macrophage metalloelastase, is a key protein secreted by macrophages that primarily targets elastin and plays crucial roles in neutrophil infiltration, cytokine release, macrophage recruitment, and proliferation during chronic inflammation.^[^ ^13^, ^14^, ^15^ ^]^ MMP12 has been implicated in promoting the invasiveness of gliomas and nasopharyngeal carcinomas. It mediates interactions between prostate cancer cells and bone marrow stromal cells during bone metastasis.^[^ ^8^ ^]^ On this basis, we hypothesized that MMP12 functions as a downstream target of SRC‐1‐mediated macrophage reprogramming in PNI. Therefore, SRC‐1 may influence pancreatic cancer PNI by regulating MMP12 secretion from TAMs. To validate this hypothesis, we transiently transfected MMP12 cDNA into siSRC‐1‐TAM cells to restore MMP12 expression (Figure 5G,H). Our results indicated that MMP12 overexpression rescued the tumor PNI capability induced by siSRC‐1‐TAMs (Figure 5J,K). MMP12 overexpression also rescued the defects in inducing tumor proliferation, migration, and invasion by siSRC‐1‐TAMs (Figure 5I,L,M,N,O). These findings further support our hypothesis that SRC‐1 regulates pancreatic cancer PNI by modulating MMP12 secretion by TAMs.

### 2.6 SRC‐1, Functioning As a Coactivator Alongside STAT1, Facilitates the Activity of the MMP12 Promoter

**Figure 6:**
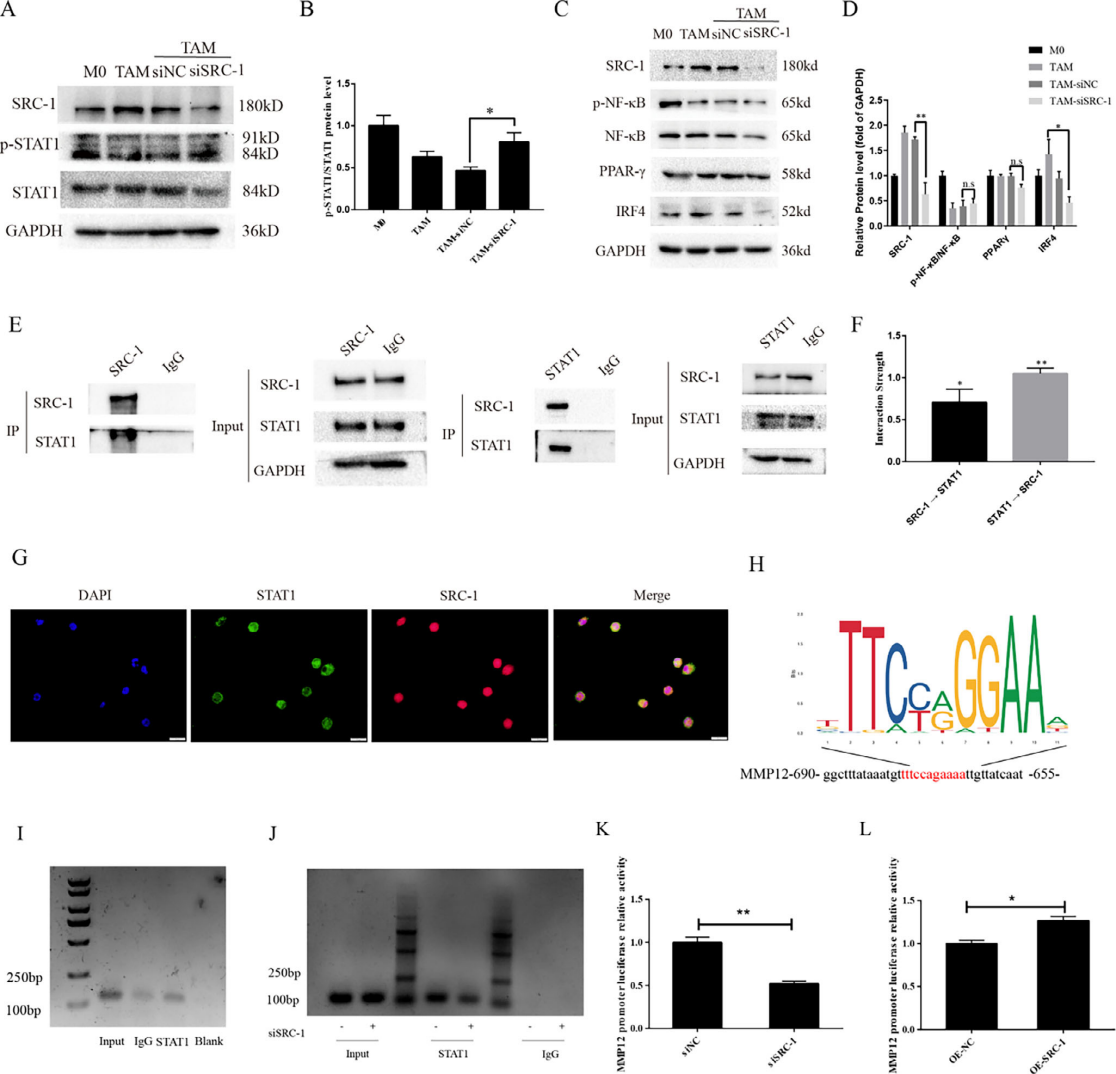
SRC‐1 as a coactivator of STAT1, promotes MMP12 promoter activity. A, B) Western blot analysis of p‐STAT1 and STAT1 expression in TAMs under different treatments. n = 3. Significance was calculated with Student's *t*‐test. ^*^ *P<*0.05. C, D) Western blot analysis of p‐NF‐κB, NF‐κB, IRF4, and PPAR‐γ expression in TAMs under different treatments.n = 3. Significance was calculated with one‐way ANOVA with Tukey post‐test.^*^ *P<*0.05,^**^ *P<*0.01, ns, no significant difference. E, F) Co‐immunoprecipitation experiments detecting the interaction between SRC‐1 and STAT1. n = 3. Significance was calculated with Student's *t*‐test. ^*^ *P<*0.05, ^**^ *P<*0.01, ^***^ *P<*0.001. G) Immunofluorescence validation of co‐localization of STAT1 (green) and SRC‐1 (red) in TAMs. H) Prediction by Jaspar of STAT1 binding sites on the MMP12 promoter region. I, J) ChIP assay confirming recruitment of STAT1 to the MMP12 promoter region. K, L) Luciferase assays demonstrating changes in MMP12 promoter luciferase activity upon knockdown or over‐expression of SRC‐1. n = 3. Significance was calculated with Student's *t*‐test. ^*^ *P* < 0.05, ^**^ *P* < 0.01.

SRC‐1 interacts with nuclear receptors and other transcription factors to initiate transcriptional networks and regulate downstream gene expression. Research indicates that SRC‐1 associates with various transcription factors such as NF‐κB, AP‐1 (c‐Jun/c‐Fos), and STAT1, synergistically activating the transcription of target genes in cells.^[^ ^8^, ^16^ ^]^ The activation of stimulus‐specific transcription factors in the macrophage genome may determine macrophage polarization by influencing the induction of gene promoters. MMP12 can cleave IFN‐γ, reducing macrophage JAK‐STAT1 signaling and diminishing the polarization of IFN‐γ‐activated macrophages.^[^ ^17^ ^]^ Therefore, we speculated that the decrease in MMP12 induced by SRC‐1 knockdown, along with the reprogramming of the macrophage polarization state by SRC‐1, also occurred through the transmission of the STAT1 signaling pathway. Furthermore, we investigated whether STAT1, a transcription factor, interacts with MMP12. Our results demonstrated that SRC‐1 knockdown increased STAT1 phosphorylation (**Figure** **6**A,B). We also examined other pathways related to macrophage polarization and found that after downregulating SRC‐1, phosphorylated NF‐κB, and peroxisome proliferator‐activated receptor gamma (PPARγ) showed no significant changes; however, interestingly, interferon regulatory factor 4 (IRF4) was downregulated (Figure 6C,D). This result suggests that SRC‐1‐mediated alterations in macrophage polarization are relatively likely to occur through the STAT1 and IRF4 pathways. We observed a similar relationship in the study by Wang et al.^[^ ^18^ ^]^found that knocking down the IRF4 gene significantly reversed the increased STAT1 phosphorylation level in LPS‐exposed BV‐2 cells. Given the established functional crosstalk among SRC‐1, IRF4, and STAT1, we propose that SRC‐1 may regulate interferon‐mediated signaling, a mechanistic insight that warrants further exploration in subsequent studies. Co‐IP analysis confirmed the physical interaction between SRC‐1 and STAT1 (Figure 6E,F). Immunofluorescence analysis confirmed the colocalization of SRC‐1 and STAT1 in macrophages (Figure 6G). JASPAR‐based prediction identified STAT1 binding sites in the MMP12 promoter region (Figure 6H), and chromatin immunoprecipitation (ChIP) experiments verified STAT1 enrichment at this locus (Figure 6I). Notably, STAT1 enrichment at the MMP12 promoter was significantly reduced in SRC‐1‐knockdown cells compared to that in controls, indicating that SRC‐1 is essential for STAT1 recruitment (Figure 6J). To assess whether SRC‐1 acts as a transcriptional coactivator of STAT1 to drive MMP12 expression, we constructed an MMP12 promoter‐driven luciferase reporter. Dual‐luciferase assays revealed that both the knockdown and overexpression of SRC‐1 altered the transcriptional activity of the MMP12 promoter (Figure 6K,L). Collectively, these results indicate that SRC‐1 interacts with STAT1 and functions as a coactivator to promote the transcription of its target gene, MMP12.

### 2.7 Inhibition of MMP12 can Effectively Alleviate PNI Both In Vivo and In Vitro

**Figure 7:**
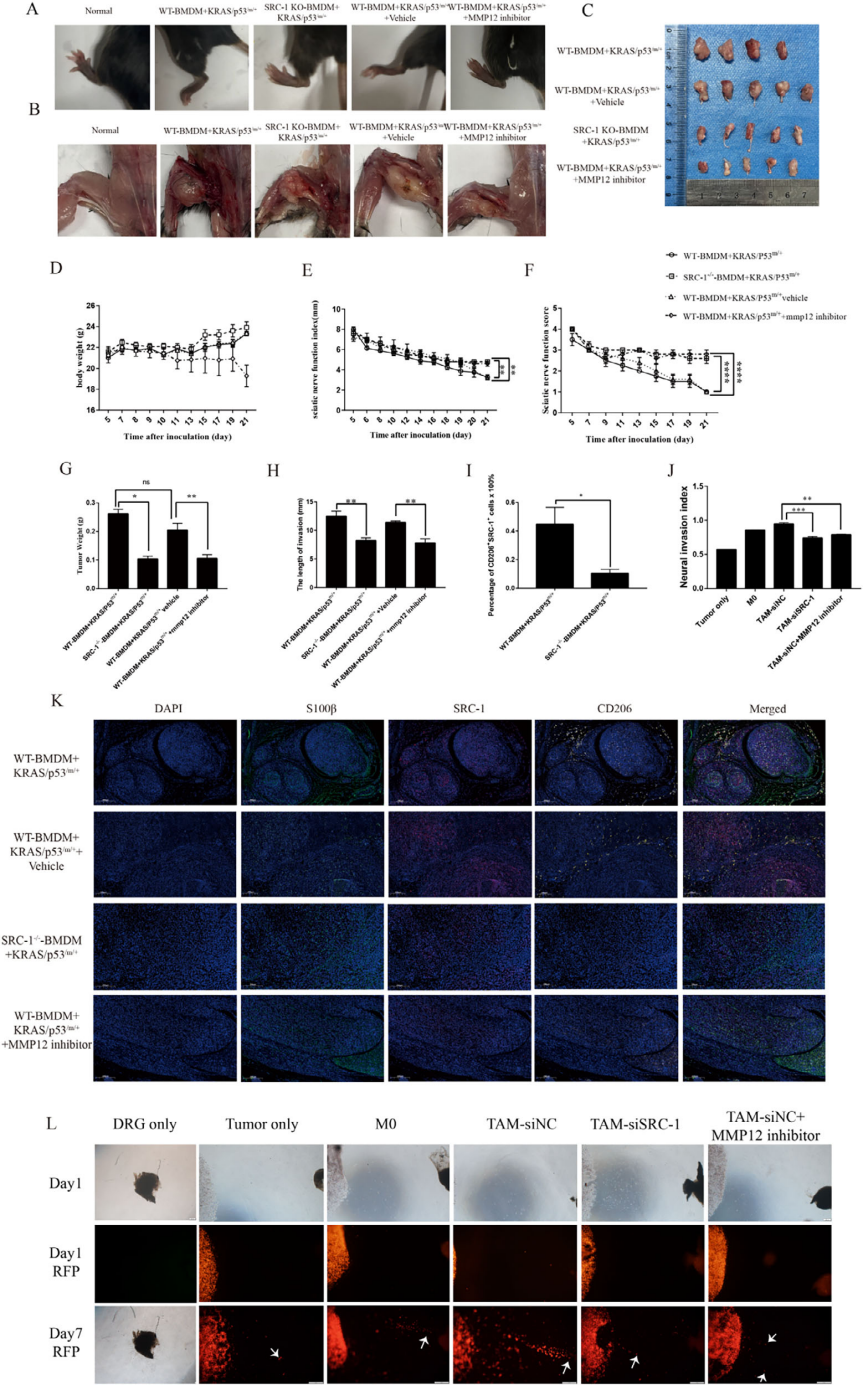
Inhibition of MMP12 can effectively alleviate PNI both in vivo and in vitro. A, B) Paralysis severity in the hind limbs of mice from various groups following sciatic nerve co‐injection of tumor and macrophage mixtures. Groups: (1) WT BMDM, (2) SRC‐1‐KO BMDM, (3) WT BMDM + vehicle, and (4) WT BMDM + MMP408 (5 mg/kg). n = 19 C) Tumor growth and invasion in the sciatic nerve of mice were recorded. D) Body weight changes in mice over a 21‐day period following sciatic nerve co‐injection of tumor and macrophage mixtures were recorded. E) Sciatic functional index in mice over 21 days following co‐injection of tumor and macrophage mixtures was recorded. WT‐BMDM+KRAS/p53^/m/+^ group: n = 4, the other groups:n = 5. Significance was calculated with one‐way ANOVA with Tukey post‐test at the final time point.^**^ *P<*0.01. F) Sciatic functional scores in mice over 21 days following co‐injection of tumor and macrophage mixtures were recorded. WT‐BMDM+KRAS/p53^/m/+^ group:n = 4, the other groups:n = 5. Significance was calculated with one‐way ANOVA with Tukey post‐test at the final time point.^****^ *P<*0.0001. G) Tumor weight in mice harvested at 21 days post‐injection was recorded. WT‐BMDM+KRAS/p53^/m/+^ group:n = 4, the other groups:n = 5. Significance was calculated with one‐way ANOVA with Tukey post‐test. ^*^ *P<*0.05, ^**^ *P<*0.01, ns, no significant difference. H) The length of invasion in the mouse groups was recorded. WT‐BMDM+KRAS/p53^/m/+^ group:n = 4, the other groups:n = 5. Significance was calculated with one‐way ANOVA with Tukey post‐test. ^**^ *P<*0.01. I) Quantitative analysis of CD206^+^ SRC‐1^+^ dual‐positive cells was performed in murine tissue using dual‐immunofluorescence staining.WT‐BMDM+KRAS/p53^/m/+^ group:n = 4, the other groups:n = 5. Significance was calculated with Student's *t*‐test. ^*^ *P<*0.05. J) Neural invasion index was recorded in the DRG‐tumor cells co‐culture assay. WT‐BMDM+KRAS/p53^/m/+^ group:n = 4, the other groups:n = 5. Significance was calculated with one‐way ANOVA with Tukey post‐test.^**^ *P<*0.01,^***^ *P<*0.001. K) Multiplex immunofluorescence co‐staining demonstrating expression of SRC‐1 (red), CD206 (yellow), and S100β (green) in murine tumor tissues. L) A DRG and tumor cell (red) co‐culture model was utilized to quantitatively assess tumor cell PNI capacity ex vivo.

To confirm the role of SRC‐1 in TAM reprogramming and the effect of MMP12 on PNI, we established four distinct animal experimental groups: (1) WT BMDM, (2) SRC‐1^‐/‐^ BMDM, (3) WT BMDM + vehicle controls, and (4) WT BMDM + MMP12 inhibitors. These BMDM populations were co‐injected with murine pancreatic cancer cells into the sciatic nerves of mice. To evaluate the extent of PNI, we monitored hind limb paralysis in mice after tumor injection. The results showed that, compared with the WT and WT + vehicle groups, the SRC‐1^‐/‐^ and MMP12 inhibitor groups exhibited significantly weaker paralysis, indicating milder nerve impairment (**Figure** **7**A). We further quantified the sciatic nerve functional status and PNI severity using the sciatic functional index and sciatic functional scores. Consistent with the paralysis findings, the indices and scores were significantly higher in the SRC‐1^‐/‐^ and MMP12 inhibitor groups than in the controls (WT and WT+vehicle groups) (Figure 7E,F), suggesting more optimized preservation of sciatic nerve function and reduced PNI severity. Additionally, measurements of tumor migration distance within nerves revealed (Figure 7H) that the SRC‐1^‐/‐^ and MMP12 inhibitor groups had shorter migration distances than the controls. Quantitative analysis of CD206 (yellow) ‐SRC‐1 (red) co‐expression via dual‐immunofluorescence revealed a decrease in dual‐positive TAMs in SRC‐1^‐/‐^ mice compared to WT controls, establishing SRC‐1 as a key regulator of TAM reprogramming in vivo (Figure 7K,I). Moreover, in the ex vivo DRG and tumor cell (red) coculture model, MMP12 inhibitor treatment attenuated PNI progression (Figure 7L,J), indicating that both SRC‐1 knockout and MMP12 inhibition effectively alleviated the severity of PNI.

**Figure 8:**
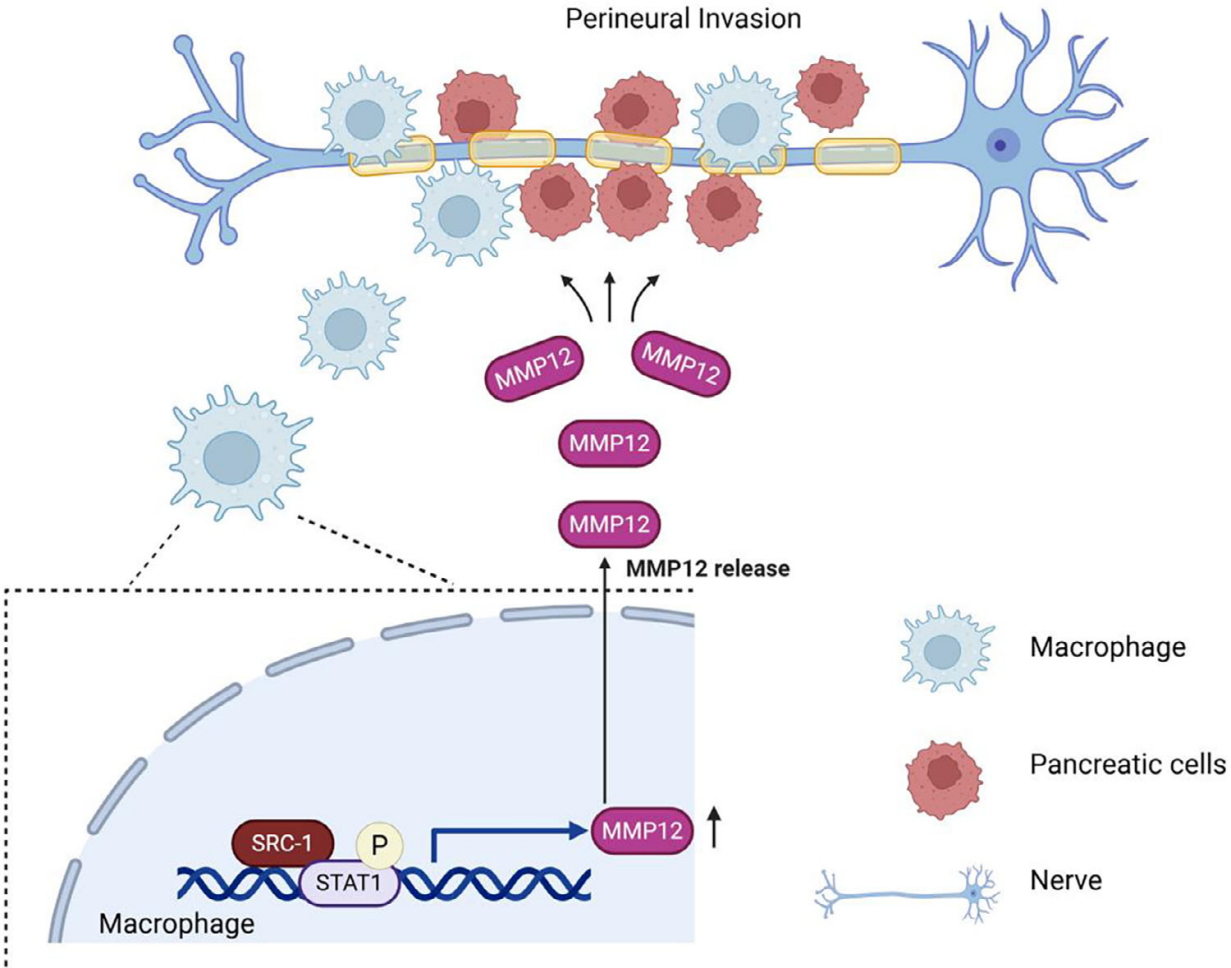
Schematic illustrating working model for the mechanisms by which SRC‐1 drives TAMs reprogramming by mediating MMP12 transcription in pancreatic cancer PNI. SRC‐1 binds to STAT1 to enhance the transcriptional activity of the MMP12 promoter, thereby increasing MMP12 secretion from TAMs.

## 3 Discussion

### 3.1 TAMs and PNI

PNI refers to the infiltration of cancer cells into nerves, affecting the pathological characteristics of malignant tumors, specifically when cancer cells approach nerves and surround at least 33% of the nerve circumference or invade any layer of the nerve membrane.^[^ ^19^ ^]^ PNI is a crucial determinant of cancer recurrence and prognosis.^[^ ^2^ ^]^ The current research indicates that TAMs play a pivotal role in the development of PNI.^[^ ^3^, ^20^, ^21^ ^]^ However, the precise role of the TAM phenotypes in PNI remains unclear. During PNI, macrophages remain active in nerves infiltrated by tumors and exhibit various polarization states. This raises two important questions: (1) What factors lead to changes in the phenotypes of macrophages recruited during PNI? (2) What roles do macrophages play in the microenvironment surrounding the nerves? This study indicated that SRC‐1 reprogrammed macrophages by activating STAT1 phosphorylation. Building on previous research, we further elucidated the specific processes of PNI, where macrophages are recruited to PNI sites and polarized into TAMs. Subsequently, SRC‐1 reduces the phosphorylation of STAT1 in TAMs, reprogramming more macrophages into TAMs, and influencing the transcriptional activity of MMP12 in TAMs. Ultimately, the reprogrammed macrophages secrete MMP12, which coordinates the invasion of cancer cells into nerves. We confirmed that PNI involves interactions between TAMs, tumor cells, and nerve cells, suggesting that interventions targeting TAM phenotypes and MMP12 secretion may represent potential strategies for preventing PNI in patients with pancreatic cancer.

### 3.2 SRC‐1 and Transcriptional Activity Regulation of MMP12

Our previous study showed that knockout of the SRC‐1 gene in mice inhibited cancer metastasis without affecting the formation of primary tumors.^[^ ^9^ ^]^ Similarly, deletion of SRC‐1 in mice reduced the response of blood vessels to estrogen‐induced damage.^[^ ^22^ ^]^ SRC‐1 interacts with p300/CREB‐binding protein, coactivator‐associated arginine methyltransferase 1(CARM1), and protein arginine N‐methyltransferase 1 (PRMT1) ^[^ ^23^, ^24^, ^25^ ^],^ collectively activating various nuclear receptors including estrogen receptor(ER), progesterone receptor (PR), retinoid X receptor(RXR), hepatocyte nuclear factor 4(HNF4), and PPARγ.^[^ ^26^ ^]^ In addition, SRC‐1 interacts with other transcription factors to function. Interactions between SRC‐1 and AP‐1, serum response factor, and NF‐κB have been reported in vitro.^[^ ^27^ ^]^ SRC‐1 plays a role in tumor progression and metastasis by modulating the transcriptional activity of key genes.^[^ ^28^ ^]^ Fundamentally, SRC‐1 serves as a major initiator of transcriptional networks, controlling effector genes and allowing cancer cells to evade treatment and ultimately metastasize to distant organs. Recruitment of SRC‐1 to AP‐1, which interacts with the colony‐stimulating factor (CSF), attracts more macrophages to the tumor site.^[^ ^29^ ^]^ Studies have indicated that SRC‐1 upregulates PD‐L1 expression, promoting immune escape of colorectal cancer cells in vitro and in vivo.^[^ ^30^ ^]^ However, whether SRC‐1 interacts with specific transcription factors to regulate MMP12 expression has not been investigated. Whether SRC‐1, a transcriptional coactivator, plays a crucial role in the regulation of MMP12 transcription remains unclear. We explored SRC‐1′s transcriptional network at different levels to investigate whether SRC‐1 also enhanced interactions with other cells in the tumor micro‐environment, such as TAMs, and whether these interactions contributed to enhancing PNI.

STAT1, one of the seven members of the STAT family, acts as a signaling molecule and transcription factor in cellular responses to cytokines and growth factors.^[^ ^31^ ^]^ Although interactions of SRC‐1 with STAT3, STAT5, and STAT6 have been previously reported, this is the first description of a STAT1/SRC‐1 interaction in macrophages. STAT3 and STAT5 are often oncogenic during tumorigenesis, whereas STAT1 is considered a tumor suppressor.^[^ ^32^ ^]^ In the current study, we found that SRC‐1 reprogrammed TAMs to participate in the regulation of pancreatic cancer PNI both in vitro and in vivo. As a coactivator of STAT1, SRC‐1 promotes MMP12 transcription, suggesting that SRC‐1 may be an attractive therapeutic target for PNI in pancreatic cancer.

Although NF‐κB is a well‐established regulator of macrophage function, our pathway screening and ChIP validation identified STAT1 as the dominant transcription factor through which SRC‐1 controls MMP12 transcription. This specificity aligns with reports that MMP12 directly cleaves IFN‐γ to modulate STAT1 signaling ^[^ ^17^ ^],^ suggesting a feed‐forward loop unique to the STAT1‐MMP12 axis in PNI. While TAMs drive tumor progression through established pathways such as CSF1‐mediated recruitment^[^ ^33^ ^]^ or IL‐10‐induced immunosuppression ^[^ ^4^ ^]^, our discovery of the SRC‐1/STAT1/MMP12 axis revealed a distinct mechanism specific to PNI. MMP12 secretion at nerve‐tumor interfaces degrades the neural ECM (e.g., elastin^[^ ^34^ ^]^), facilitating cancer cell invasion along axons. In contrast with cytokine‐focused pathways, SRC‐1 directly co‐activates STAT1 to regulate MMP12 transcription, creating a feed‐forward loop where MMP12 cleavage of IFN‐γ further modulates STAT1 signaling.^[^ ^17^ ^]^ This contrasts with the role of SRC‐1 in breast cancer metastasis via M‐CSF1^[^ ^33^ ^]^ and TWIST ^[^ ^29^ ^],^ which highlights its context‐dependent functions.

### 3.3 Key Novel Aspects of Our Work

PNI‐specific mechanism: We demonstrated that SRC‐1 reprogrammed TAMs to secrete MMP12, directly facilitating cancer cell invasion along the nerves. This axis is distinct from that described in previous reports on the role of SRC‐1 in general metastasis or macrophage polarization.

STAT1/MMP12 transcriptional regulation: We, for the first time, provide evidence that SRC‐1 binds to STAT1 to activate the MMP12 promoter, a pathway critical for PNI, but this has not been explored in other contexts.

In vivo validation: Using sciatic nerve injection models, we showed that SRC‐1‐deficient TAMs attenuate PNI progression, highlighting their therapeutic relevance in pancreatic cancer.

Our research mainly explored the regulatory role of SRC‐1 in the polarization status of macrophages and its influence on the secretion of enzymes by macrophages. Specifically, the polarization status of macrophages regulated by SRC‐1 changes, and the secretion of MMP‐12 by these macrophages changes accordingly, thereby further affecting the biological characteristics of tumor cells and altering the interaction between the tumor and nerve cells (i.e., PNI). This mechanism reveals the dynamic changes in communication and interaction between different cell types, which is fundamentally different from previous studies that focused solely on the function of SRC‐1 in tumor cells.

### 3.4 Therapeutic Targeting of SRC‐1 holds Promise But is Limited

SRC‐1 inhibitors can be combined with immunotherapy to overcome resistance to PD‐1/PD‐L1 inhibitors.^[^ ^30^ ^]^ In colorectal cancer models, combination therapy with an SRC‐1 inhibitor and PD‐L1 antibody resulted in tumor regression rates increasing more than two‐fold compared to monotherapy, accompanied by a three‐fold increase in CD8⁺ T‐cell infiltration.^[^ ^30^ ^]^ Notably, our findings suggest that the inhibition of SRC‐1 may serve as a therapeutic target for mitigating PNI in pancreatic cancer. Pancreatic tumors exhibit a dense stroma, which limits drug penetration. Nanoparticles or macrophage‐targeted carriers may improve the bioavailability of SRC‐1 inhibitors.

However, the development of SRC‐1 inhibitors remains challenging. For instance, cytokines in the tumor microenvironment (e.g., IL‐6) may activate STAT3 through bypass pathways, thereby compromising the efficacy of SRC‐1 inhibitors.^[^ ^30^ ^]^

### 3.5 Continuity and Limitations of the Study

Our previous study demonstrated that exosomes secreted by pancreatic cancer cells carry XIST, which can be transferred to nerve cells to induce GDNF secretion, thereby influencing the process of pancreatic cancer PNI.^[^ ^35^ ^]^ However, the PNI microenvironment involves more than just the tumor and nerve cells; other components are involved. Therefore, this study focused on the role of TAMs in this process. Interestingly, we previously discovered a close relationship between SRC‐1 and XIST in glioma studies ^[^ ^36^ ^],^ and we have now identified a connection between SRC‐1 and PNI. Therefore, we hypothesized that there may be a relationship between SRC‐1, XIST, and PNI, which will be the focus of our future research.

Our study had several limitations. Although we focused on TAMs, SRC‐1‐mediated PNI likely involves crosstalk with other stromal cells, such as neurons and fibroblasts. In our previous study, cancer‐derived exosomes carrying XIST^[^ ^35^ ^]^ may activate neurons to secrete GDNF. This may synergize with SRC‐1 mediated TAM‐secreted MMP12. Cancer‐associated fibroblasts (CAF) also drive PNI in pancreatic cancer.^[^ ^37^ ^]^ Enhanced glycolysis in CAFs drives the formation of a high‐lactate tumor microenvironment that favors cancer progression.^[^ ^37^ ^]^ Coincidentally, in an acetaminophen‐induced liver injury model, the intercellular crosstalk between MMP12⁺ macrophages was found to be sustained by enhanced glycolysis.^[^ ^38^ ^]^ This observation led us to speculate that the SRC‐1‐mediated secretion of MMP12 from macrophages may also be associated with glycolytic reprogramming in CAF‐mediated PNI. Hence, future studies could explore (1) neuron‐TAM co‐cultures with SRC‐1 modulation; (2) single‐cell RNA‐seq of PNI niches to map cellular interactions; and (3) CAF and TAM interactions during PNI.

Based on our experimental results, the downregulation of SRC‐1 in TAMs was accompanied by the corresponding downregulation of IRF4. This observation prompted us to consider that SRC‐1 may also have a functional connection with interferon regulatory factors, which warrants further investigation. In future investigations, we will focus on elucidating the relationship between SRC‐1 and interferon signaling, extending our research to multiple disease models to determine whether their interaction represents a broad biological phenomenon. Finally, although SRC‐1 is expressed in diverse cell types, our functional studies (e.g., macrophage‐specific knockout and MMP12 rescue) demonstrated that its role in TAMs is indispensable for PNI. Future studies should explore whether SRC‐1 contributes to the PNI in neurons or whether stromal cells contribute to PNI through alternative mechanisms.

## 4 Conclusion

In summary, this study identified SRC‐1 as a critical regulator of TAM reprogramming, which drives PNI in pancreatic cancer. Multiple mechanisms are presented in (**Figure** **8**). We demonstrated that SRC‐1 binds to STAT1 to enhance the transcriptional activity of the MMP12 promoter, thereby increasing MMP12 secretion from TAMs. The SRC‐1/STAT1/MMP12 axis promotes TAM polarization toward a pro‐tumor M2 phenotype, facilitates cancer cell proliferation, migration, and invasion, and ultimately accelerates PNI progression. Genetic ablation of SRC‐1 in TAMs and pharmacological inhibition of MMP12 significantly attenuated PNI in both in vitro and in vivo models. Our findings revealed a novel mechanistic link between macrophage reprogramming and neural invasion, positioning the SRC‐1/STAT1/MMP12 axis as a promising therapeutic target for mitigating PNI in pancreatic cancer.

## 5 Experimental Section

### Tissue Microarray

Human pancreatic cancer tissue microarrays were obtained from Hunan Aifang Biotechnology Co., Ltd. Pancreatic cancer on tissue microarrays was classified according to the diagnosis by experienced pathologists. The tissue microarrays contained 44 samples of which 16 were diagnosed with severe neural invasion, and the remaining 28 showed no significant neural involvement. Immunofluorescence staining was scored on a four‐point scale (scores 0–3). Scores of 2 or 3 were regarded as high expression, and scores of 0 or 1 were regarded as low expression. Using the VisloPharm Pathology Quantitative Analysis software, the tissue surrounding the nerves was selected for a 200 × field‐of‐view screenshot. The expression of various fluorescent signals in the cells was read, positive signals were selected, and the color selection standard was saved. The “Multiple Phenotyping” feature was utilized for classification and grading, displaying the target and calculating the number of CD68‐positive cells and the fluorescent area of SRC‐1. The study using the tissue microarray was approved by the Life Sciences Ethics Committee of Changsha Yaxiang Biotechnology Co., LTD. The Ethics report is available online at yxswll.ccrl.cn. The query code is 15LC4G78LR30G0. The approval number is Csyayj2024089.

### Cell Culture

The human pancreatic cancer cell lines PANC‐1 and ASPC‐1; human monocytic leukemia cell line THP‐1; and human kidney epithelial cell line 293T were purchased from the American Type Culture Collection (ATCC). Professor Paul J. Chiao provided the mouse pancreatic cancer cell line KRAS/p53^m/+^ from the University of Texas MD Anderson Cancer Centre. PANC‐1, ASPC‐1, 293T, and KRAS/p53^m/+^ cells were cultured in Dulbecco's modified medium (DMEM) containing 10% fetal bovine serum (FBS) and 1% penicillin‐streptomycin. THP‐1 cells were cultured in RPMI 1640 medium supplemented with 10% FBS, 1% penicillin‐streptomycin, and 0.05 mm β‐mercaptoethanol.

For small RNA interference knockdown experiments, the CALNP RNAi reagent (D‐Nano Therapeutics, DN001‐05) was used according to the manufacturer's instructions. In the overexpression experiments, all transient transfections were conducted using GoldenTran‐DR (DR14926015) following the instructions provided in the product manual.

Mouse primary bone marrow‐derived macrophages (BMDMs) were isolated from 8–10‐week‐old wild‐type and SRC‐1 knockout mice. After euthanizing the mice by cervical dislocation, the limbs were disinfected with 75% ethanol, and the muscle tissues were removed to expose the femur and tibia. The ends of the bones were trimmed, and 5 ml of DMEM was injected into the marrow cavities of the femur and tibia to flush out the bone marrow, which was collected in a 15 ml centrifuge tube. This flushing was repeated twice until the bone became colorless. The cell suspension was then centrifuged, the supernatant was discarded, and 3 ml of red blood cell lysis buffer was added to disperse the cell pellet. After 20 s, an equal volume of the complete medium was added to neutralize the reaction. The suspension was centrifuged again, the supernatant was discarded, and the cells were resuspended in DMEM supplemented with 10% FBS and 1% penicillin‐streptomycin. The cells were plated in culture dishes and incubated at 37 °C with 5% CO_2_. After the cells adhered, 20 ng/ml M‐CSF was added, and the medium was changed daily until day 7 to obtain BMDMs.

### Preparation of Conditioned Medium (CM)

When ASPC‐1 cells or the indicated groups of TAM cells reached 90% confluence, the medium was replaced with serum‐free medium, and the cells were cultured for 48 h. The conditioned medium was collected and centrifuged at 2000 rpm for 10 min to remove the cell pellets. The supernatant was mixed with fresh complete medium at a 1:1 ratio to prepare the CM.

### Induction and Treatment of THP‐1 Cells

THP‐1 cells were treated with PMA (Sigma, P1585, 100 nm) for 24 h. The medium was replaced with conditioned media (CM) from pancreatic cancer cells and stimulated for 48 h to obtain tumor‐associated macrophages (TAM).

### Western Blot (WB)

The protein lysis buffer was prepared, and the protein was quantified using a BCA protein assay kit (TIANGEN, PA115). Then, the appropriate volume of loading buffer was added, and the mixture was boiled at 100 °C for 10 min. SDS gels were prepared at varying concentrations according to the experimental requirements. The samples were loaded sequentially as required, and the gel was run at 80V for the stacking gel and 120V for the separating gel. Wet transfer was performed at 350 mA for 1.5 h. The membrane was blocked with 5% non‐fat dry milk at room temperature(25 °C) for 1.5 h. Primary antibodies were diluted according to recommended ratios: SRC‐1 (CST, 2191, 1:1000), CD206 (Proteintech, 60 143, 1:5000), CD163 (WANLEI, WL03026, 1:1000), iNOS (Proteintech, 22 226, 1:1000), MMP12 (Proteintech, 22 989, 1:500), STAT1 (CST, 9172, 1:1000), p‐STAT1 (CST, 7649, 1:1000), and GAPDH (Proteintech, 10 494, 1:5000). The cells were incubated overnight at 4 °C. Secondary antibodies were selected based on the source of the primary antibodies: Anti‐rabbit IgG (7074, 1:2500; CST) and Anti‐mouse IgG (7076, 1:2500; CST). The mixture was then incubated at room temperature for 1 h. The bands were developed using ECL chemiluminescence reagent.

### Hematoxylin and Eosin (H&E) Staining

The tissue sections were baked at 60 °C for 2 h to ensure proper adhesion. Sections were dewaxed and rehydrated with water. The sections were stained with hematoxylin for 1 min, followed by differentiation using hydrochloric acid alcohol for 1 s. The sections were rinsed in running water, and the color was checked under a microscope. If the color was unsuitable, the staining and differentiation steps were repeated until the desired color was achieved. Then, bluing was performed for 20 min in running water. The sections were immersed in 85% alcohol for 1 min, then stained with eosin for 3 min. The sections were rinsed in running water, and the color was checked. The sections were dehydrated and mounted with neutral balsam. Sections were air‐dried in a well‐ventilated area, observed, and photographed under a microscope.

### Immunohistochemical Staining

The tissue sections were incubated at 60 °C for 2 h. Sections were dewaxed and rehydrated with water. Staining was performed according to the instructions provided with the immunohistochemistry kit (ZSGB‐Bio, PV9000). The dilution ratios of the primary antibodies were as follows: S100 (Proteintech, 15 146, 1:200), CK19 (Proteintech, 10 712, 1:6000), CD68 (Abcam, ab955, 1:3000), and SRC‐1 (CST, 2191, 1:200).

### Immunofluorescence Staining

After baking the slices at 60 °C for 2 h for tissue sections, deparaffinization was performed until the sections were fully immersed in water, followed by heat‐induced antigen retrieval. After discarding the cell supernatant and washing thrice with PBS, the cells were fixed with 4% paraformaldehyde for 15 min, followed by washing with PBS to remove any residual paraformaldehyde. Subsequently, both the tissue sections and cells were permeabilized with 0.25% Triton X‐100 for 15 min, followed by three washes with PBS. The samples were blocked with goat serum at 37 °C for 30 min. After removal of the blocking solution, the first antibody was added and incubated overnight at 4 °C. The following day, the sections were washed with PBS and incubated with the corresponding secondary antibodies at room temperature for 1 h. After washing with the secondary antibody, the samples were stained with DAPI for 5 min at room temperature. After a final wash with PBS to remove excess DAPI, the samples were sealed with an anti‐fade mounting medium. Ki67 (Proteintech, 27 309, 1:200), F4/80 (Abcam, ab111101, 1:50), CD206 (Proteintech, 60 143, 1:200), STAT1 (Proteintech, 66 545, 1:200), SRC‐1 (CST, 2191, 1:200).

### Enzyme‐Linked Immunosorbent Assay (Elisa)

CM was collected from the TAM group. ELISA kits (Jiangsu Meibiao Biological Technology, MB‐3705B, MB‐0122B, MB‐2868B, MB‐2912B) were used according to the manufacturer's instructions. The plates were removed from the aluminum foil bag after equilibration at room temperature for 20 min. Standard and sample wells were established; then 50 µL of different concentrations of the standard solution was added to the standard wells. For the sample wells, 10 µL of the sample to be tested was added, followed by the addition of 40 µL of the sample dilution buffer (resulting in a 5‐fold dilution). No samples were added to the blank wells. Except for the blank wells, 100 µL of HRP‐conjugated detection antibody to each of the standard and sample wells. The wells were sealed with a plate‐sealing film and incubated at 37 °C in a water bath or incubator for 60 min. The liquid was discarded, and the wells were blotted with absorbent paper. Sufficient wash buffer was added to each well, allowed to sit for 1 min, after which the wash buffer was discarded and the wells were blotted dry again. This washing step was repeated 5 times. Then 50 µL of substrate A and 50 µL of substrate B were added to each well, and incubated at 37 °C in the dark for 15 min, followed by the addition of 50 µL of stop solution to each well. Within 15 min, the OD density of each well was measured at 450 nm.

### Co‐Culture of Tumor Cells and Dorsal Root Ganglia

Six to eight‐week‐old C57BL/6 mice were selected, euthanized by cervical dislocation, and sterilized using 75% ethanol. The dorsal skin was cut open, the muscles were removed, the spine was extracted, and then cut along the sagittal plane. The dorsal root ganglia were isolated and trimmed under a microscope. The dorsal root ganglia were fixed at one end of a 12‐well plate using 5 µL of matrix gel and incubated for 30 min until the gel solidified. Meanwhile, the tumor cells were digested, and the cell concentration was adjusted to 3 × 10^4^, then the cells were dissolved in 10 µL of matrix gel. This mixture was seeded on the other side of the same well ≈2–3 mm next to the DRG. The distance between the dorsal root ganglia and tumor cells was maintained consistently across the wells. Once the matrix gel had solidified, the appropriate culture medium was added and incubated. The MMP12 inhibitor group was treated with MMP408 (80 nm). The medium was renewed every other day. The neural invasion index was recorded through observations and photographs. The neural invasion index was calculated as α/β. Total Distance (β) was regarded as the linear distance between the tumor cell mass centroid and the nearest DRG edge (µm). Invasion Distance(α) was regarded as the longest linear distance migrated by tumor cells toward DRG, measured from the tumor mass leading edge to the foremost invading cell nucleus. All measurements were performed independently by two blinded investigators. Measurements were taken on day 7 of co‐culture.

### CCK8 Assay

After treating the ASPC‐1 cells with different concentrations of TAM‐CM for 48 h, equal numbers of cells were seeded into a 96‐well plate. After another 48 h, the CCK8 reagent was added, and the absorbance was measured at 450 nm.

### Colony Formation Assay

After treating ASPC‐1 cells with different concentrations of TAM‐CM for 48 h, equal numbers of cells were seeded in a 6‐well plate. After ≈1 week, the plates were removed and stained with crystal violet, and the number of cell colonies was counted.

### Wound Healing Assay

After treating the ASPC‐1 cells with different concentrations of TAM‐CM for 48 h, equal numbers of cells were seeded into a 12‐well plate. Once the cells had adhered, the pipette tip was scratched. After 48 h, cell migration was observed.

### Animal Experiment

Six to eight‐week old C57 mice were used for the wild‐type (WT) and SRC‐1 knockout (SRC‐1^‐/‐^) groups. Bone marrow‐derived macrophages (BMDM) were extracted and cultured for 7 days. After stimulation with the CM from KRAS/p53^m/+^ mouse pancreatic cancer cells, BMDM was induced to transform into TAM. Five six to eight‐week old C57 mice were selected, with 3 × 10^4^ KRAS/p53^m/+^ cells and 1 × 10^5^ WT‐BMDM injected into the left sciatic nerve, and 3 × 10^4^ KRAS/p53^m/+^ cells and 1 × 10^5^ SRC‐1^‐/‐^ BMDM injected into the right sciatic nerve. MMP12 inhibitor MMP408 (Sigma, 444 291) was administered via daily oral gavage (5 mg/kg in 200 µl PBS) from day 4 to 21 post‐tumor injection. Disinfection and observation were conducted daily, with a 5‐day recovery period for nerve injury. Starting on the sixth day post‐injection, the body weight, sciatic nerve function score (SFS), and sciatic nerve function index (SFI) were recorded every other day. The SFS reflected the response of the hindlimb to manual extension, with a score of 4 indicating normal function, 3 indicating mild impairment, 2 indicating severe impairment, and 1 indicating complete paralysis. The SFI was measured as the length between the first and fifth toes in their natural extension, expressed in millimeters (mm).^[^ ^35^ ^]^ All animal selections and groupings were randomly generated through a draw to ensure no bias or unintended influence from researchers. To avoid subjective bias in the study of the sciatic nerve functional index and sciatic nerve functional scoring, the researchers involved in the scoring were unaware of the specific groups and treatment information of the mice. All animal experimental procedures followed the guidelines of the Ethics Committee of Dalian Medical University. C57BL/6 mice were obtained from the Liaoning Changsheng Biotechnology Co., Ltd. The approval number for animal experiments: CSE202404002.

### Protein Identification by Liquid Chromatography–Mass Spectrometry (LC‐MS)

Three six to eight‐week‐old WT and three SRC‐1‐/‐ C57 mice were used to extract primary BMDM. After culturing in DMEM supplemented with 10 ng/ml macrophage‐colony stimulating factor for 7 days, the BMDM were stimulated with CM from KRAS/p53m/+ mouse pancreatic cancer cells to induce their transformation into TAM. Following a 48‐h incubation in serum‐free medium, the culture supernatants were collected, centrifuged to remove dead cells, and analyzed via LC‐MS to identify differential proteins.

### Real‐Time Quantitative PCR

After removing the culture medium, the cells were washed with PBS. An appropriate amount of TRIZOL was added to lyse the cells, and the sample was transferred to a nuclease‐free centrifuge tube. Then the supernatant was centrifuged and discarded. Chloroform was added, mixed well, and allowed to stand before centrifugation was repeated. Then the upper transparent liquid was transferred to a new centrifuge tube. Isopropanol was then added, mixed well, and allowed to stand before centrifugation. The supernatant was discarded, and the gel‐like pellet was retained at the bottom. The pellet was washed with 75% alcohol, air‐dried, and dissolved in DEPC pyrocarbonate to obtain RNA. Reverse transcription was performed using an RNA reverse transcription kit (Vazyme, R223‐01), and real‐time quantitative PCR was conducted using ChamQ Universal SYBR qPCR Master Mix (Vazyme, Q711) according to the manufacturer's instructions for the reaction system and conditions.

### Immunoprecipitation (IP)

Immunoprecipitation (IP) was performed using magnetic beads (Santa Cruz, SC‐2003) and according to the manufacturer's instructions. Then SRC‐1 (CST, 2191, 1:100) and STAT1 (CST, 9172, 1:50) were detected.

### Chromatin Immunoprecipitation (ChIP)

The ChIP assay was performed according to the manufacturer's instructions, using a ChIP kit (Abclonal, RK20258) and magnetic beads (Santa Cruz, 500 775). STAT1 (CST, 9172, 1:50).

### Dual‐Luciferase Assay

A Dual‐Luciferase Assay Kit (Promega, E1910) was used to perform the luciferase assay according to the manufacturer's instructions.

### Statistical Analysis

All quantitative data were pre‐processed by normalization to internal controls (GAPDH for Western blot). All quantitative data were presented as mean ± Standard Error of Mean (SEM). For in vitro studies, sample sizes were n = 3 for all groups. For in vivo experiments, sample sizes were n = 5 for all groups, with the exception of the WT‐BMDM+KRAS/p53^m/+^ group, which had n = 4. Statistical significance was calculated by Student's *t*‐test, Fisher's exact test, and ANOVA. *p* value < 0.05 was regarded as a statistically significant difference. Statistical analyses were performed using GraphPad Prism.

## Conflict of Interest

The authors declare no conflict of interest.

## Data Availability

The data that support the findings of this study are available from the corresponding author upon reasonable request.
